# Effects of synergistic tongue and chin resistance training on swallowing function, oral intake, and cognitive function in community-dwelling elderly individuals with frailty: a double-blind randomised controlled trial

**DOI:** 10.7189/jogh.15.04358

**Published:** 2025-11-05

**Authors:** Yen-Fang Chou, Kondwani Joseph Banda, Ruey Chen, Chien-Mei Sung, Kai-Jo Chiang, Li-Fang Chang, Pi-Yu Su, Kuei-Ru Chou

**Affiliations:** 1 School of Nursing, College of Nursing, Taipei Medical University, Taipei, Taiwan; 2 Department of Nursing, New Taipei Municipal TuCheng Hospital (Built and Operated by Chang Gung Medical Foundation), New Taipei, Taiwan; 3 Department of Gerontology and Health Care Management, Chang Gung University of Science and Technology, Taoyuan, Taiwan; 4 Department of Surgery, Kamuzu Central Hospital, Lilongwe, Malawi; 5 Department of Nursing, Taipei Medical University-Shuang Ho Hospital, New Taipei, Taiwan; 6 Post-Baccalaureate Program in Nursing, Taipei Medical University, Taipei, Taiwan; 7 College of Nursing, National Defense Medical University, Taipei, Taiwan; 8 Department of Nursing, Tri-Service General Hospital, Taipei, Taiwan; 9 Department of Medical Education, Taipei Medical University Hospital, Taipei, Taiwan; 10 Graduate Institute of Medical Sciences, College of Medicine, National Defense Medical University, Taipei, Taiwan; 11 Department of Nursing, Wan Fang Municipal Hospital, Taipei, Taiwan; 12 Research Center in Nursing Clinical Practice, Wan Fang Municipal Hospital, Taipei, Taiwan; 13 Psychiatric Research Center, Taipei Medical University Hospital, Taipei, Taiwan; 14 Research Center for Neuroscience, Taipei Medical University, Taipei, Taiwan

## Abstract

**Background:**

Tongue strengthening exercises (TSE) and chin tuck against resistance (CTAR) improve swallowing function. However, previous findings are limited to post-stroke population, single-mode therapies, and immediate post-test assessment only, while evidence on effects of combined therapies in community-dwelling elderly individuals with frailty is unknown. Therefore, we explored effects of synergistic TSE and CTAR on swallowing function (tongue strength, swallowing pressure, tongue endurance, and lip strength), oral intake, and cognitive function.

**Methods:**

A prospective three-arm parallel-group double-blind randomised controlled trial conducted in community residential care facilities. Participants were assigned to TSE + CTAR (n = 31), CTAR (n = 30), or control group: cheek-bulging exercises (n = 30) by block randomisation with block size set at six and sealed opaque envelopes used for allocation concealment. Swallowing training included two phases – (i) initial swallowing training (baseline to 3-month) and (ii) a 3-month booster training initiated immediately after T4 (6-month follow-up) – conducted for 30-minute/session, 3-sessions/d, 6 days/week for 3 months. Outcomes were assessed at 7 time points: baseline, 1-month mid-test, 2-month mid-test, 3-month post-initial training test, 6-month, 9-month, and 12-month follow-up. Data were analysed using generalised estimating equations (GEE) with group, time, and group × time interaction as fixed effects under an intention-to-treat framework.

**Results:**

Ninety-one community-dwelling elderly individuals with frailty (mean age 83.4 ± 6.9 years; 77% women) were enrolled. Significant group × time interactions were observed for tongue strength: anterior tongue strength (ATS) (β = 6.5, 95% CI = 1.6–11.4) and posterior tongue strength (PTS) (β = 8.4, 95% CI = 3.0–13.7) and swallowing pressure saliva swallowing pressure (SSP) (β = 13.3, 95% CI = 8.5–18.2) and effortful swallowing pressure (ESP) (β = 6.2, 95% CI = 0.7–11.7) immediately post-test, with sustained improvements at 9-month and12-month following booster training. Chin tuck against resistance alone produced similar but smaller improvements in tongue strength: ATS (β = 7.8, 95% CI = 3.2–12.4) and PTS (β = 7.0, 95% CI = 2.4–11.5) and swallowing pressure: SSP (β = 13.4, 95% CI = 8.3–18.5), and ESP (β = 8.0, 95% CI = 2.1–13.9) immediate post-test, with sustained improvement at 9-month and 12-month following booster training. Although trends toward better tongue endurance, lip strength, oral intake, and cognitive function were observed, these changes were not statistically significant.

**Conclusions:**

Synergistic TSE + CTAR, reinforced by booster training, produced statistically significant yet moderate improvement in swallowing function, especially tongue strength and swallowing pressure, compared to CTAR or cheek-bulging exercises alone.

**Registration:**

Chinese Clinical Trial Registry Identifier: ChiCTR2400091807.

Globally, the increase in the population of older adults (≥65 years) has been accompanied by a rise in the incidence of frailty [1,2]. Frailty is a geriatric syndrome characterised by decreased physiological reserves, sarcopenia, muscle weakness, and heightened vulnerability to external stressors [2,3]. These age-related declines compromise neuromuscular and functional integrity, positioning frailty as a major risk factor for impaired swallowing function, which is a critical determinant of nutritional status, aspiration risk, and overall health in older adults [4]. Previous evidence has shown a strong association between frailty and swallowing dysfunction [5–9] with dysphagia affecting nearly one-third of community-dwelling older adults [10]. Consequently, the management of dysphagia-related complications in frail elderly individuals should be encouraged using targeted and integrated swallowing rehabilitation strategies.

Tongue strengthening exercises (TSE) [11,12] and chin tuck against resistance (CTAR) [13,14] are established swallowing rehabilitation strategies. The tongue strengthening exercises (TSE) involves tongue protrusion, elevation, and lateralisation against resistance, which enhances tongue muscle strength and coordination essential for bolus manipulation and propulsion [11,12,15]. This is relevant for frail older adults, where tongue weakness and sarcopenia of oropharyngeal muscles are common contributors to swallowing dysfunction [7,10]. Similarly, CTAR targets the suprahyoid muscles responsible for laryngeal elevation and airway protection, thereby promoting safe and efficient swallowing [13,14,16,17]. Evidence suggests that CTAR effectively improves swallowing biomechanics but could also reduce risk of aspiration in frail individuals [8,18]. Given the complex relationship between frailty, sarcopenia, and dysphagia [5,9], TSE and CTAR would represent vital components of comprehensive dysphagia management in community-dwelling elderly individuals with frailty.

Recent evidence demonstrates that TSE [12,15] and CTAR [16,17] significantly improve swallowing function in adults. However, several important gaps remain in the current literature. First, most studies have been conducted in patients with post-stroke dysphagia [16,17], limiting generalisability to community-dwelling elderly individuals with frailty. Second, majority of trials have focused on single-mode therapies, with limited investigation of combined swallowing interventions [11,12,17,19]. Third, few studies have examined long-term efficacy, as most report immediate post-intervention outcomes [11,17]. Combining TSE and CTAR provides a multidimensional approach that targets both oral and pharyngeal phases of swallowing, thereby offering more comprehensive improvement and enhancing quality of life in frail older adults. To address the current research gap, we conducted a double-blind, randomised controlled trial to evaluate the effects of synergistic TSE and CTAR on swallowing function (tongue strength, swallowing pressure, tongue endurance, and lip strength), oral intake, and cognitive function in community-dwelling elderly individuals with frailty.

## METHODS

### Study design and participants

This prospective, three-arm, parallel-group, double-blind randomised controlled trial [20] was conducted at residential care facilities in Taiwan and enrolled community-dwelling elderly individuals with frailty. Eligible participants were screened through structured interviews and functional assessments conducted by trained research nurses. Those meeting inclusion criteria and providing written informed consent were consecutively enrolled. An independent researcher, who was not involved in participant recruitment, intervention, or assessment, performed block randomisation [21] with block size set at six, ensuring balanced group assignment and minimising selection bias, consistent with established methodological guidance. Allocation concealment was maintained using sequentially numbered, opaque, sealed envelopes prepared by the independent researcher. Participant assignment was implemented by a research assistant (Wang) [22], while a research nurse (Chang) was responsible for administering the designated intervention by opening the sealed envelopes sequentially.

The study rigorously implemented and maintained double-blinding throughout the entire study period. Participants, trainers, and outcome assessors were blinded to group allocation. Participants were informed only that they would receive one of several validated exercise programmes for swallowing health, without being told which represented the experimental or control condition. Trainers followed standardised instructional scripts and pre-recorded video protocols to maintain uniformity and minimise expectancy effects. Outcome assessors (for IOPI, FOIS, and MMSE) and the statistical analyst remained blinded to allocation, and communication between assessors and trainers was strictly restricted to prevent unblinding. These procedures ensured that double-blinding was successfully maintained, despite the behavioural nature of the intervention, thereby minimising performance and detection bias in accordance with CONSORT guidelines. Data collection occurred between 1 August 2020 and 31 July 2023, and the trial was registered with the Chinese Clinical Trial Registry (Identifier: ChiCTR2400091807).

The study inclusion criteria included:

(i) age ≥65 years;

(ii) at least one point of frailty on the Fried Frailty Assessment Scale;

(iii) subjective memory decline;

(iv) and ability to communicate in Mandarin or Taiwanese.

The exclusion criteria included:

(i) severe communication difficulties;

(ii) inability to exercise independently or reliance on caregiver assistance;

(iii) inability to lift the head or neck;

(iv) current use of anticholinergics, benzodiazepines, or antihistamines;

(v) diagnosis of aspiration pneumonia;

(vi) presence of a tracheostomy;

(vii) and neuromuscular disorders or dementia.

Before screening, written informed consent was obtained from all participants. Baseline assessments included demographic characteristics (age, sex, socioeconomic status, marital status, and chronic diseases), activities of daily living, instrumental activities of daily living, swallowing function, frailty status, nutritional status, and mood (**Table 1****;** Table S1 in the **Online Supplementary Document**).

**Table 1:** Demographic characteristic of the participants at baseline (n = 91)*

Variable	G0	G1	G2	Total	F/χ^2^	*P-*value
	**(n = 30)**	**(n = 31)**	**(n = 30)**	**(n = 91)**		
**Sex, n (%)†**					χ^2^_(2)_ = 0.46	0.797
Male	7 (23.3)	6 (19.4)	8 (26.7)	21 (23.1)		
Female	23 (76.7)	25 (80.6)	22 (73.3)	70 (76.9)		
**Age (years)‡**					F _(2,88)_ = 0.21	0.815
Range (MD)	70–95 (83)	72–94 (83)	67–101 (83)	67–101 (83)		
Mean (SD)	83.83 (7.53)	82.77 (5.66)	83.67 (7.59)	83.42 (6.91)		
**Marital status, n (%)§**					4.71	0.587
Single	1 (3.3)	0 (0.0)	2 (6.7)	3 (3.3)		
Married	11 (36.7)	15 (48.4)	9 (30.0)	35 (38.5)		
Widowed	17 (56.7)	16 (51.6)	18 (60.0)	51 (56.0)		
Divorced	1 (3.3)	0 (0.0)	1 (3.3)	2 (2.2)		
**Education, n (%)§**					2.55	0.882
≦Primary school	4 (13.3)	8 (25.8)	6 (20.0)	18 (19.8)		
Middle school	5 (16.7)	3 (9.7)	5 (16.7)	13 (14.3)		
High school	9 (30.0)	9 (29.0)	10 (33.3)	28 (30.8)		
≧College school	12 (40.0)	11 (35.5)	9 (30.0)	32 (35.2)		
**Religious, n (%)§**					7.46	0.441
No	7 (23.3)	3 (9.7)	5 (16.7)	15 (16.5)		
Buddhism	10 (33.3)	16 (51.6)	13 (43.3)	39 (42.9)		
Taoism	0 (0.0)	2 (6.5)	0 (0.0)	2 (2.2)		
Christianity	11 (36.7)	10 (32.3)	11 (36.7)	32 (35.2)		
Catholicism	2 (6.7)	0 (0.0)	1 (3.3)	3 (3.3)		
**Living status, n (%)†**					χ^2^_(2)_ = 0.26	0.879
Alone	23 (76.7)	22 (71.0)	22 (73.3)	67 (73.6)		
Not alone	7 (23.3)	9 (29.0)	8 (26.7)	24 (26.4)		
**Comorbidities type‡**					F _(2,88)_ = 1.52	0.223
Range (MD)	1–7 (3)	1–6 (2)	1–8 (3)	1–8(3)		
Mean (SD)	3.30 (1.60)	2.81 (1.62)	3.57 (1.49)	3.22 (1.74)		
**Take medicine§**					2.21	0.392
No	1 (3.3)	2 (6.5)	4 (113.3)	7 (7.7)		
Yes	29 (96.7)	29 (93.5)	26 (86.7)	84 (92.3)		
**Family history of dementia§**					0.22	1.000
No	28 (93.3)	29 (93.5)	28 (93.3)	85 (93.4)		
Yes	2 (6.7)	2 (6.5)	2 (6.7)	6 (6.6)		
ADL(BI)‡	96.0 (6.62)	97.4 (4.63)	93.7 (11.81)	95.7 (8.29)	F _(2,88)_ = 1.612	0.205
IADL‡	7.2 (1.01)	6.9 (1.15)	6.2 (2.49)	6.8 (1.72)	F _(2,88)_ = 3.211	0.050
MNA‡	25.8 (3.22)	27.0 (1.71)	26.7 (2.21)	26.5 (2.48)	F _(2,88)_ = 2.053	0.135
GDS‡	2.7 (2.91)	1.5 (1.65)	1.6 (1.81)	1.9 (2.23)	F _(2,88)_ = 2.675	0.075
MMSE‡	25.5 (3.25)	25.4 (2.33)	24.5 (5.47)	25.1 (3.88)	F _(2,88)_ = 0.655	0.558

G0 – control group, G1 – experimental group 1 received tongue strengthening exercises (TSE) and chin tuck against resistance exercise training (TSE + CTAR), G2 – experimental group 2 received chin tuck against resistance exercise training (CTAR), ADL – activities of daily living, IADL – instrumental activities of daily living, MNA – mini-nutritional assessment, GDS – geriatric depression scale, MD – median, MMSE – mini-mental state examination

*Data are presented as mean ± standard deviation or frequency (%).

†χ^2^ test.

‡One-way ANOVA.

§Fisher exact test.

### Sample size

The sample size was calculated using G*Power 3.1, employing F-tests for MANOVA with repeated measures and within-between interaction [23]. Based on a priori effect size of η^2^ = 0.40 [17], α = 0.05, power = 0.80, three groups, and seven repeated measurements, the estimated total sample size was 60 (20 participants per group). Allowing for a 20% dropout rate exceeding the 12% reported in prior research [24] and oversampling to enhance statistical power, the final target sample size was set at 90 (approximately 30 per group). The assumed large effect size (η^2^ = 0.40) was based from findings of Park et al. (2019) [17], who reported a large-to-very-large effect (Cohen’s f = 0.85; η^2^ = 0.42) for CTAR-based resistance swallowing training. Given the comparable nature of the intervention, adopting a similar effect size was deemed appropriate for the current study. To minimise attrition bias and preserve randomisation integrity, the intention-to-treat (ITT) principle was employed in the statistical analysis.

### Intervention

The study implemented a structured two-phase 3-month swallowing training programme incorporating tongue strengthening exercises (TSE) [11,12,15] and chin tuck against resistance (CTAR) [13,14,16,17], adapted from prior literature and clinical practice to meet the needs of frail, community-dwelling older adults. Swallowing training programme was delivered in two consecutive 3-month phases:

(i) initial training (T0–T3)

(ii) and booster training (T4–T5), initiated immediately after the 6-month follow-up (T4).

**Figure 1:**
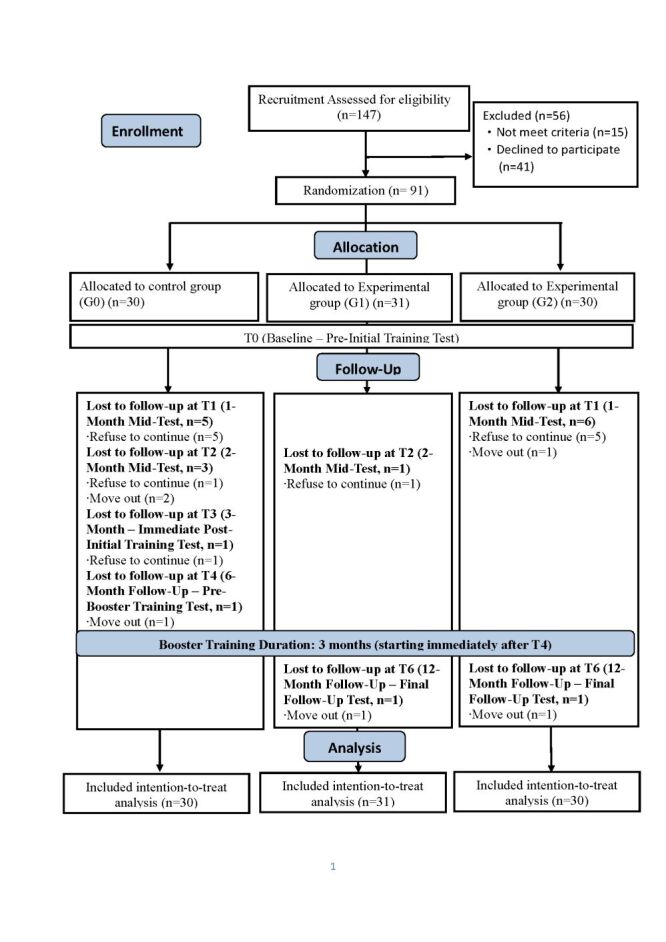
CONSORT flow diagram for participant enrolment.

**Figure 2:**
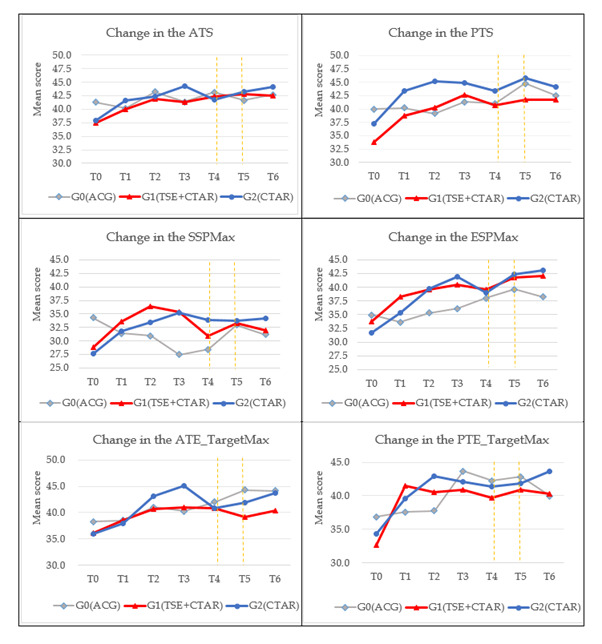
Change scores of the outcomes at baseline to 12-month follow-up following booster training.

**Figure 3:**
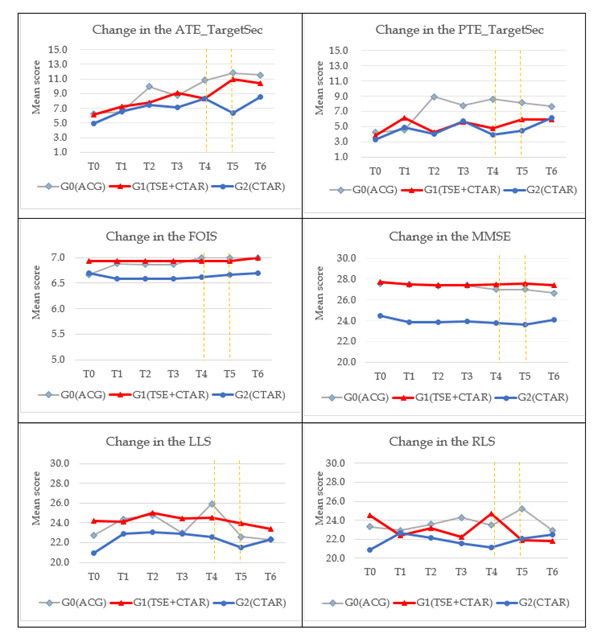
Change scores of the outcomes at baseline to 12-month follow-up following booster training.

In both phases, swallowing training protocol was delivered 30-minute/session, 3 times/d, and 6 days/week for 3 months. Outcomes were assessed at seven time points: T0 (baseline – pre-initial training), T1 (1-month mid-test), T2 (2-month mid-test), T3 (3-month immediate post-initial training), T4 (6-month follow-up – Pre-Booster Training Test), booster training starting immediately after T4, T5 (9-month follow-up – Immediate Post-Booster Training Test), and T6 (12-month follow-up – Final Follow-Up Test) (**Figure 1**, **Figure 2**, **Figure 3**). To ensure prevention of deviation from intended interventions, trainers provided instructional videos and communication software. Adherence was monitored via daily logs, weekly phone checks, and periodic video review. Participants were permitted to have rest breaks in case of fatigue or intercurrent illness which were recorded.

#### Experimental Group 1 (TSE+CTAR)

Participants performed a combined protocol targeting both tongue and suprahyoid musculature. The TSE training (15 minutes) included five exercises designed to enhance tongue strength, mobility, and coordination:

(i) posterior sweep,

(ii) protrusion,

(iii) retraction,

(iv) lateralisation,

(v) and tongue-palate press.

Each TSE exercise was performed with maximum effort, held for 1–3 seconds, then relaxed and repeated. The CTAR training (15 minutes) included isometric and isotonic tasks using a small rubber ball. The isometric exercise involved pressing the chin against the ball for 10 seconds, repeated 10 times, while the isotonic exercise involved 10 repetitions of dynamic chin tuck motions against the ball. The TSE+CTAR protocol was the same across both initial and booster training phases.

#### Experimental Group 2 (CTAR only)

Participants performed CTAR exclusively for 30 minutes, divided equally between isometric (15 minutes) and isotonic (15 minutes) exercises, following the same protocol as described above. The CTAR protocol was the same across both initial and booster training phases.

#### Active control group (Cheek-Bulging Exercises)

Participants performed cheek-bulging exercises for 30 minutes. The protocol involved inflating the cheeks, expanding them outward for 5 seconds before relaxing, followed by alternately blowing air into the right and left sides of the mouth to maintain facial muscle activation while avoiding direct engagement of swallowing musculature and reduce expectancy bias. The cheek-bulging exercises protocol was the same across both initial and booster training phases.

### Outcome measures

#### Primary outcome indicator: swallowing function

The primary outcome was swallowing function measured by:

(i) tongue strength [25,26],

(ii) swallowing pressure [27],

(iii) tongue endurance [25,26],

(iv) and lip strength [28,29] measured by Iowa Oral Performance Instrument (IOPI) [30–34].

The Iowa Oral Performance Instrument (IOPI) (IOPI Medical, Redmond, WA, USA) is a portable handheld device widely used for objective assessment of swallowing function. The device consists of a pressure-sensitive rubber bulb, which records peak pressures in kilopascals (kPa) [31,35]. For each parameter, participants perform three trials with 1-minute rest intervals, and the highest value is recorded to ensure reliability and minimise intra-individual variability [32,36,37].

#### Secondary outcome indicators: oral intake and cognitive function

Secondary outcomes included oral intake and cognitive function. Oral intake was measured by the Functional Oral Intake Scale (FOIS) [38]. Cognitive function was measured by the Chinese Mini-Mental State Examination (MMSE) [39–41].

Oral intake was assessed using the 7-level FOIS. The FOIS classifies patients’ functional eating ability, where higher scores reflect greater oral intake and improved swallowing function [38]. The FOIS has demonstrated excellent psychometric properties, including high interrater reliability (κ = 0.86–0.91) and strong validity (r = 0.90) [38].

Cognitive function was evaluated with the Chinese Mini-Mental State Examination (MMSE) [39–41], which examines seven cognitive domains (orientation, registration, attention and calculation, recall, language, and visuospatial ability) with a maximum score of 30. A score below 24 was considered indicative of cognitive impairment, further classified as mild (21–24), moderate (10–20), or severe (≤9). The MMSE has been validated in Chinese populations, showing good diagnostic performance with sensitivity of 85% and specificity of 82% for detecting cognitive impairment [39–41].

All assessments were performed by trained outcome assessors blinded to group allocation. To ensure measurement consistency and minimise assessor-related variability, all outcome assessors underwent structured inter-rater reliability training and standardisation procedures before participant enrolment. The training was coordinated by two senior speech-language pathologists and one neuropsychologist, each with more than 10 years of clinical experience. The calibration process included theoretical instruction, live demonstrations, and repeated pilot assessments to achieve consistent administration of all instruments. Specifically, outcome assessors were trained to perform IOPI-based measurements (tongue pressure and endurance), FOIS (Functional Oral Intake Scale), and MMSE (Mini-Mental State Examination) using standardised testing scripts that specified verbal cues, coaching effort level, bulb placement position, and rest intervals. Inter-rater reliability was established using intraclass correlation coefficients (ICCs) from double-rated pilot data, showing excellent agreement: ICC = 0.93 for IOPI, ICC = 0.90 for FOIS, and ICC = 0.95 for MMSE. During the trial, all measurements were performed with the same model of IOPI device, which was calibrated weekly according to manufacturer specifications to ensure accuracy across time and sites.

### Statistical analysis

Data analyses were conducted using IBM SPSS Statistics for Windows, Version 25.0 (IBM Corp., Armonk, NY, USA). Baseline differences among groups were examined using one-way analysis of variance (ANOVA) for continuous variables [42], the Kruskal-Wallis test for ordinal data [43], and χ^2^ or Fisher exact tests for categorical variables [44]. Generalized Estimating Equations (GEE) were employed [45] to evaluate the longitudinal effects of synergistic TSE and CTAR interventions on swallowing function (tongue strength, swallowing pressure, tongue endurance, and lip strength), oral intake, and cognitive function across seven assessment points (T0–T6). The statistical analysis comprised five sequential steps addressing: model specification, handling of repeated measures, robustness and inference, handling of missing data, and post-hoc comparisons to ensure comprehensive and transparent evaluation of longitudinal effects.

#### Model specification

For continuous outcomes (assumed to follow a normal distribution), GEE models were fitted using an identity link function. Each model included Group, Time, and the Group × Time interaction as fixed effects, with participant ID specified as the clustering variable. The primary inference regarding treatment efficacy was derived from the Group × Time interaction term, representing differential change trajectories between groups over time.

#### Handling of repeated measures

An exchangeable working correlation structure was adopted to model within-subject correlations, assuming equal correlation across all time points within each participant. Model adequacy was assessed by comparing QIC values across exchangeable, independence, and autoregressive structures, with the exchangeable model providing the best fit.

#### Robustness and inference

All inferences were based on Wald Type III tests, and robust (sandwich) standard errors were applied to ensure consistency even in cases of potential correlation structure misspecification.

#### Handling of missing data

Analyses followed the intention-to-treat (ITT) principle. Missing observations were addressed using GEE’s maximum-likelihood estimation under the missing-at-random (MAR) assumption, complemented by multiple imputation (20 data sets) as a sensitivity analysis. Consistent results across these approaches indicated that missing data had minimal influence on the overall findings.

#### Post-hoc comparisons

When a significant Group × Time interaction was identified, post-hoc pairwise comparisons were conducted to determine specific time points exhibiting between-group differences. The Least Significant Difference (LSD) method was applied to adjust for multiple comparisons. All tests were two-tailed, with all statistical significance set at α = 0.05.

## RESULTS

### Participants characteristics

A total of 147 community-dwelling older adults were screened for eligibility; 56 were excluded (15 did not meet inclusion criteria and 41 declined to participate). Finally, 91 participants (mean age = 83.4 ± 6.9 years), of whom 70 were women (76.9%) were randomised into three groups. At 12 months, 84 participants (92.3%) completed follow-up assessments. All analyses adhered to the intention-to-treat (ITT) principle, and missing data were imputed using multiple imputation procedures (**Figure 1**). Most participants were widowed (56.0%) and had a college/university degree (35.2%). Most of the participants lived alone (73.6%) with an average number of chronic diseases of 3.2 (SD = 1.8), and 84 participants (92.3%) on medication for chronic conditions. Baseline demographic characteristics did not differ significantly across the three groups (**Table 1**; Table S1 in the **Online Supplementary Document**). Overall, adherence rates ranged from 60% in the TSE + CTAR group, 57% in CTAR group, and 45% in control group. No serious adverse events occurred during the study. These results indicate that, despite the programme’s intensity, the swallowing training programme was both feasible and safe for community-dwelling elderly individuals with frailty when implemented under supervision and tailored to individual tolerance.

### Effects of synergistic TSE and CTAR on the primary outcome

The primary outcome was swallowing function, comprising:

(i) tongue strength,

(ii) swallowing pressure,

(iii) tongue endurance,

(iv) and lip strength.

Tongue strength included anterior (ATS) and posterior (PTS); swallowing pressure included saliva (SSP) and effortful (ESP); tongue endurance included Target Max (ATE/PTE) and Target Sec (ATE/PTE); lip strength included left (LLS) and right (RLS). Across domains, group × time interactions were significant for tongue strength (ATS, PTS) and swallowing pressure (SSP, ESP), indicating greater improvements in the intervention arms vs control, with benefits maintained after the booster phase at 9-month and 12-month. Endurance and lip strength showed no significant between-group differences over time.

#### Tongue strength (ATS/PTS)

TSE + CTAR demonstrated significant group × time effects on ATS at immediate post-test (β = 6.5, 95% CI = 1.6–11.4), and remained increased at 9-month (β = 6.8, 95% CI = 1.9–11.8) and 12-month (β = 5.7, 95% CI = 1.1–10.2) follow-up compared to the control group. CTAR alone demonstrated significant group × time effects on ATS at immediate post-test (β = 7.8, 95% CI = 3.2–12.4) and 9-month follow-up (β = 6.9, 95% CI = 1.2–12.6) compared to the control group (**Figure 2**; Table S2–3 in the **Online Supplementary Document**).

TSE + CTAR demonstrated significant group × time effects on PTS at immediate post-test (β = 8.4, 95% CI = 3.0–13.7) and 6-month (β = 7.6, 95% CI = 2.1–13.0) and 12-month (β = 7.3, 95% CI = 2.3–12.2) follow-up compared to the control group. CTAR alone also demonstrated significant group × time effects on PTS at immediate post-test (β = 7.0, 95% CI = 2.4–11.5) compared to the control group (**Figure 2**; Table S2 and S4 in the **Online Supplementary Document**).

#### Swallowing pressure (SSP/ESP)

TSE + CTAR demonstrated significant group × time effects on SSP at immediate post-test (β = 13.3, 95% CI = 8.5–18.2) and 6-month (β = 8.6, 95% CI = 2.8–14.5), 9-month (β = 6.5, 95% CI = 0.9–12.8) and 12-month (β = 7.2, 95% CI = 1.5–12.8) follow-up compared to the control group. CTAR alone also demonstrated significant group × time effects on SSP at immediate post-test (β = 13.4, 95% CI = 8.3–18.5) compared to the control group (**Figure 2**; Table S2 and S5 in the **Online Supplementary Document**).

TSE + CTAR demonstrated significant group × time effects on ESP at immediate post-test (β = 6.2, 95% CI = 0.7–11.7) and 12-month (β = 6.8, 95% CI = 0.5–13.1) follow-up. CTAR alone also demonstrated significant group × time effects on ESP at immediate post-test (β = 8.0, 95% CI = 2.1–13.9) (**Figure 2**; Table S2 and S6 in the **Online Supplementary Document**).

#### Tongue endurance (Target Max/Target Sec)

Target Max (ATE/PTE) & Target Sec (ATE/PTE): no significant group × time effects were detected. Point estimates favoured Synergistic TSE + CTAR and CTAR alone at selected time points, but the overall trajectories were not different for the experimental and control groups (**Figure 2**, **Figure 3****;** Table S2, S7–S10 in the **Online Supplementary Document**).

#### Lip strength (LLS/RLS)

No significant between-group differences over time were observed for LLS and RLS. Lip strength remained stable across follow-up (**Figure 3****;** Table S2, S11–12 in the **Online Supplementary Document**).

### Effects of synergistic TSE and CTAR on the secondary outcome

The secondary outcomes were oral intake measured by Functional Oral Intake Scale (FOIS) and cognitive function measured by Chinese Mini-Mental State Examination (MMSE).

#### Oral intake

No significant between-group differences were found for FOIS scores at any time point (*P* > 0.05). Within-group comparisons indicated stable oral intake performance across experimental and control groups (**Figure 3**; Table S2 and S13 in the **Online Supplementary Document**).

#### Cognitive function

No significant between-group differences were found for MMSE scores at any time point (*P* > 0.05). Although synergistic TSE + CTAR showed a slight upward trend after booster training, the difference was not statistically significant (**Figure 3**; Table S2 and S14 in the **Online Supplementary Document**).

## DISCUSSION

This randomised controlled trial demonstrated that synergistic TSE + CTAR and CTAR only produced statistically significant yet moderate improvements in tongue strength (ATS, PTS) and swallowing pressure (SSP, ESP) among community-dwelling older adults with frailty. In contrast, tongue endurance, lip strength, oral intake, and cognition did not show meaningful changes across follow-up. These findings highlight the selective benefits of resistance-based oropharyngeal training, while acknowledging its limited scope of improvement. At 6-month follow-up, a decline in effects suggested possible detraining, while booster training restored and maintained gains through nine to 12 months, emphasising that periodic reinforcement is essential for sustaining swallowing performance.

### Synergistic TSE and CTAR and swallowing function – tongue strength and endurance

The findings demonstrated that synergistic TSE + CTAR significantly improved tongue strength, including anterior (ATS) and posterior tongue strength (PTS), with effects sustained for up to 12 months in community-dwelling elderly individuals with frailty. These results are consistent with prior studies [15,46], supporting the potential of structured strength-based training for long-term neuromuscular adaptation. The synergistic benefit of TSE and CTAR may be explained by their complementary mechanisms as TSE strengthens intrinsic tongue muscles and improves coordination [11,12,15], whereas CTAR activates suprahyoid muscles, facilitating hyolaryngeal excursion and enhancing swallowing force [13,14,16,17]. Improvements in tongue strength and swallowing pressure declined between post-test and 6-month follow-up, while the initiation of booster training at 6-month follow-up for an additional three months reinforced and sustained the benefits of TSE+CTAR and CTAR at 9-month and 12-month follow-up. The significant improvement in tongue strength observed at 12 months reveal the importance of continued delivery of swallowing exercises to counteract the detraining effects. These findings suggest that swallowing rehabilitation should not be limited to a single intervention period but also incorporate structured booster training to ensure long-term sustenance of functional gains for community-dwelling elderly individuals with frailty.

In contrast, tongue endurance remained stable across the study period, with no significant improvements observed at immediate post-test and follow-up. This may be attributed to the exercise protocols in this study focusing on strength development rather than prolonged muscular activity. Improvement in endurance may require repetitive, low-resistance activation of muscle fibres, whereas the short-duration isometric nature of TSE and CTAR may not provide sufficient stimulus. Prior research indicates that endurance-based, repetitive training approaches may be more effective for this outcome [47]. Since impaired tongue endurance in community-dwelling elderly individuals with frailty may compromise swallowing safety during extended meals, future studies should integrate endurance-focused rehabilitation interventions to complement strength-based interventions ensuring comprehensive rehabilitative strategy.

Our findings align with previous reports indicating that resistance-based tongue and suprahyoid training enhances swallowing pressure and oral-phase control [15,46]. Although, the observed pressure gains were modest (5–8 kPa), they exceeded the minimal clinically important difference (MCID) for tongue pressure improvement (>5 kPa) reported by Robbins et al. (2005) [48]. This magnitude of change is considered clinically meaningful, as it may contribute to improved bolus propulsion, enhanced airway protection, and reduced aspiration risk in frail older adults. Moreover, sustaining even moderate increases in tongue and swallowing pressure can support nutritional intake, shorten mealtime duration, and maintain quality of life in individuals with declining oropharyngeal function. These findings therefore provide evidence that the observed physiological improvements while moderate in size hold practical and preventive value for swallowing safety in geriatric populations.

### Synergistic TSE and CTAR and swallowing function – swallowing pressure and lip strength

The findings revealed that synergistic TSE + CTAR and CTAR alone produced significant improvement in swallowing pressure, including saliva swallowing pressure (SSP) and effortful swallowing pressure (ESP), at immediate post-test and during follow-up. These results are consistent with previous research showing that strength-based swallowing exercises enhance oral-phase swallowing function by improving bolus control and pharyngeal clearance [15,16]. Significant increase in SSP demonstrates more efficient tongue-palate contact during natural swallowing, while significant increase in ESP reveal ability to generate stronger volitional pressure during swallowing. Together, these improvements reflect better coordination of lingual and suprahyoid muscle activity, which is essential for safe and effective swallowing. Moreover, increased SSP is associated with safer swallowing during everyday meals, reduced risk of aspiration, and better airway protection, while increased ESP provides compensatory reserve that can be recruited during challenging swallowing tasks, such as processing solid foods or when fatigued. These improvements may also reduce mealtime duration, lower the risk of malnutrition and dehydration, and decrease reliance on caregiver assistance, thereby supporting independence and quality of life among community-dwelling elderly individuals with frailty. Notably, the sustained improvements observed at 12 months following booster training highlight that increase in swallowing pressure can be maintained with periodic reinforcement, highlighting the importance of long-term strength-based training strategies for community-dwelling elderly individuals with frailty.

In contrast, lip strength remained stable across the study period, with no significant improvements observed at immediate post-test and follow-up [48]. The possible explanation is that TSE and CTAR primarily target the tongue and suprahyoid muscles rather than the orbicularis oris and perioral musculature essential for lip seal. Inadequate lip strength can impair bolus control and intraoral pressure generation, indirectly compromising swallowing safety and efficiency. The absence of gains in this domain indicates that tongue-focused interventions alone are insufficient to improve lip strength. Prior studies have shown that targeted approaches, such as isometric lip-closure training and resistance-based lip strengthening exercises, can enhance perioral muscle performance [49]. However, tongue endurance and lip strength remained largely unchanged, likely because the TSE and CTAR protocols emphasised short-duration, high-intensity isometric loading, which may not sufficiently stimulate endurance-type muscle fibres or perioral musculature. Future research should integrate endurance-focused repetitive tasks and perioral resistance exercises to enhance global orofacial function [47,48]. Therefore, a multimodal rehabilitation strategy that integrates tongue, lip, and other orofacial exercises may provide a more comprehensive approach to optimising oral function and feeding independence in community-dwelling elderly individuals with frailty.

### Synergistic TSE and CTAR and oral intake and cognitive function

The study revealed that oral intake remained stable across the study period, with both TSE+CTAR and CTAR alone showing non-significant effects at immediate post-test and follow-up. However, the relatively normal baseline FOIS scores across all groups indicate that participants were functionally independent in oral intake and did not exhibit clinically significant dysphagia, thereby limiting the potential for observable improvement. Swallowing involves coordinated activation of an extensive cortical–subcortical network, including the primary motor and sensory cortices, insula, anterior cingulate, supplementary motor area, basal ganglia, and cerebellum [50–52]. In community-dwelling elderly individuals with frailty, delayed corticobulbar conduction, reduced cortical excitability, and weakened sensorimotor integration can impair swallowing efficiency without showing signs of dysphagia [50–52]. Moreover, these latent neurofunctional declines may reduce the neuroplastic potential required for adaptive cortical reorganisation following muscle-based interventions. Consequently, the stable FOIS scores observed across the study likely reflect a ceiling effect coupled with preserved compensatory cortical mechanisms that sustain functional oral intake despite age- and frailty-related neurophysiological alterations in oropharyngeal control among community-dwelling elderly individuals with frailty.

Similarly, cognitive function remained stable across the study period, with MMSE scores in the intervention groups remaining within the normal cognitive range, while the control group exhibited slightly lower scores. This finding suggests that the swallowing training interventions may have contributed to cognitive maintenance rather than enhancement among community-dwelling older adults with frailty. Such stability aligns with prior research indicating that muscle-based swallowing exercises provide limited engagement of higher-order cortical networks essential for cognitive improvement [52,53]. Frailty is characterised by cerebral hypoperfusion, white matter microstructural damage, and reduced synaptic plasticity, leading to decreased efficiency within large-scale brain networks responsible for attention, memory, and executive control [54]. The cortical regions activated during swallowing, including the insula, prefrontal cortex, and parietal areas partially overlap with those involved in cognitive control and attentional regulation, suggesting a neurophysiological pathway for oropharyngeal motor–cognitive transfer through shared circuitry [53]. However, the low cognitive demand of the present swallowing training protocol may have been insufficient to stimulate these associative cortical regions and promote measurable cognitive adaptation. Therefore, the observed stability in MMSE scores suggests preserved neurocognitive functioning, emphasising the need for integrated and combined cognitively enhanced interventions to effectively engage prefrontal and parietal circuits and promote neuroplasticity and cognitive adaptation in community-dwelling elderly individuals with frailty.

### Strengths and limitations

To the best of our knowledge, this randomised controlled trial is among the first to demonstrate the long-term effects of a combined Tongue Strengthening Exercise (TSE) and Chin Tuck Against Resistance (CTAR) programme on swallowing function in community-dwelling older adults with frailty, underscoring the importance of booster training for sustaining treatment effects over time. The adoption of a multimodal rehabilitation strategy targeting both intrinsic tongue muscles and the suprahyoid musculature represents a novel and synergistic approach to swallowing therapy. Furthermore, the inclusion of a highly vulnerable yet underrepresented population contributes valuable evidence to the fields of dysphagia rehabilitation and geriatric research. The study was methodologically rigorous conducted according to CONSORT reporting standards, guided by a registered study protocol, and analysed using an intention-to-treat framework, thereby minimising bias and enhancing internal validity.

Nonetheless, some limitations should be acknowledged. First, while the cheek-bulging exercise served as an active control designed to match attention and engagement, it may not have been a fully equivalent comparator. This task primarily activates the buccinator and facial muscles, differing from the experimental interventions that target the tongue and suprahyoid musculature, key components of swallowing biomechanics. As such, between-group differences could partly reflect distinct muscle activation patterns rather than purely intervention-specific effects. The cheek-bulging control was selected for feasibility, safety, and tolerability among frail participants, ensuring comparable contact time and minimising fatigue risk. However, future research should consider attention-matched sham interventions to more precisely isolate the neuromuscular mechanisms underlying TSE and CTAR. Second, the generalisability of the findings may be limited as the participants were cognitively intact, mildly frail, community-dwelling older adults, which may not reflect institutionalised or acutely ill populations who typically exhibit more severe dysphagia or cognitive impairment. This inclusion strategy was intentionally adopted to ensure participant safety, adherence, and accurate task execution without caregiver support. Accordingly, the results should not be generalised to individuals with moderate-to-severe cognitive impairment, advanced dysphagia, or neuromuscular disorders. Future studies should include participants with broader cognitive and functional profiles to evaluate the feasibility and efficacy of synergistic TSE + CTAR interventions across the full spectrum of frailty and dysphagia severity.

## CONCLUSIONS

The study findings demonstrate that synergistic TSE + CTAR and CTAR alone, reinforced by booster training, produced statistically significant yet moderate improvements in tongue strength and swallowing pressure compared with cheek-bulging exercises alone among community-dwelling elderly individuals with frailty. Although moderate in magnitude, these improvements exceeded the minimal clinically important difference for tongue pressure, supporting their clinical relevance in enhancing swallowing safety and potentially reducing aspiration risk in this population. The sustained effects observed following booster sessions underscore the importance of long-term reinforcement strategies to maintain neuromuscular gains over time. However, no significant changes were observed in tongue endurance, lip strength, oral intake, and cognitive function, suggesting that strength-focused interventions alone may be insufficient to yield broader functional or neurocognitive benefits. These findings highlight the need for multimodal interventions that integrate oropharyngeal strengthening with endurance-based training and the high-intensity nature of the training programme suggests that future research involving community-dwelling elderly individuals with frailty should explore reduced session frequency, progressive load adaptation, or hybrid tele consider their physical condition to optimise adherence, accessibility, and long-term sustainability.

## Additional material


Online Supplementary Document
