# Supplementary material for: Effects of synergistic tongue and chin resistance training on swallowing function, oral intake, and cognitive function in community-dwelling elderly individuals with frailty: a double-blind randomised controlled trial
Source: J Glob Health. 2025 Nov 5;15:04358. doi: 10.7189/jogh.15.04358 (PMC12679056; doi:10.7189/jogh.15.04358)
Supplement: Online Supplementary Document [file jogh-15-04358-s001.pdf]

Supplement to: Chou Y-F, Banda KJ, Chen R, Sung C-M, Chiang K-J, Chang L-F, Su P-Y, Chou K-R. Effects of synergistic tongue and chin resistance training on swallowing function, oral intake, and cognitive function in community-dwelling elderly individuals with frailty: a double-blind randomised trial. J Glob Health. 2025;15:04358.

Table S1. Demographic Characteristics of the Participants

Table S2. Effects of Synergistic TSE and CTAR on Swallowing Function (Tongue Strength, Swallowing Pressure, Tongue Endurance, and Lip Strength), Oral Intake, and Cognitive Function

Table S3. GEE Analysis of Differences between Pretest and Posttest for Anterior Tongue Strength

Table S4. GEE Analysis of Differences between Pretest and Posttest for Posterior Tongue Strength

Table S5. GEE Analysis of Differences between Pretest and Posttest for Saliva Swallowing Pressure

Table S6. GEE Analysis of Differences between Pretest and Posttest for Effortful Swallowing Pressure

Table S7. GEE Analysis of Differences between Pretest and Posttest for Anterior Tongue Endurance Target Maximum

Table S8. GEE Analysis of Differences between Pretest and Posttest for Posterior Tongue Endurance Target Maximum

Table S9. GEE Analysis of Differences between Pretest and Posttest for Anterior Tongue Endurance Target Second

Table S10. GEE Analysis of Differences between Pretest and Posttest for Posterior Tongue Endurance Target Second

Table S11. GEE Analysis of Differences between Pretest and Posttest for Left Lip Strength

Table S12. GEE Analysis of Differences between Pretest and Posttest for Right Lip Strength

Table S13. GEE Analysis of Differences between Pretest and Posttest for Oral Intake

Table S14. GEE Analysis of Differences between Pretest and Posttest for Cognitive Function.

Table S1. Demographic Characteristics of the Participants (n = 91)

Variable	G0 (n = 30) n(%)	G1 (n = 31) n(%)	G2 (n = 30) n(%)	Total (n = 91) n(%)	F/χ^2	*P* value
Comorbidities, n (%) [a]	30(100.0)	31(100.0)	30(100.0)	91(100.0)		**NA**
Types of chronic dis (multiple choices)						
Stroke	0(0.0)	0(0.0)	3(10.0)	3(3.3)		
Hypertension	20(66.7)	20(64.5)	15(50.0)	55(60.4)		
Cardiovascular disease e	7(23.3)	6(19.4)	6(20.0)	19(20.9)		
Hyperlipidemia	12(40.0)	9(29.0)	7(23.3)	28(30.8)		
Sleep disorder	15(50.0)	8(25.8)	5(16.7)	28(30.8)		
Cataract	7(23.3)	6(19.4)	12(40.0)	25(27.5)		
Diabetes mellitus	8(26.7)	5(16.1)	5(16.7)	18(19.8)		
Osteoporosis	3(10.0)	6(19.4)	7(23.3)	16(17.6)		
BPH	5(16.7)	1(3.2)	6(20.0)	12(13.2)		
MCI	1(3.3)	2(6.5)	7(23.3)	10(11.0)		
Arthritis	3(10.0)	2(6.5)	4(13.3)	9(9.9)		
Depression	1(3.3)	4(12.9)	3(10.0)	8(8.8)		
kidney disease	2(6.7)	2(6.5)	2(6.7)	6(6.6)		
Comorbidities type [b]					$F_{(2,88)} = 1.52$	0.223
Range(median)	1-7(3)	1-6(2)	1-8(3)	1-8(3)		
Mean(SD)	3.30(1.60)	2.81(1.62)	3.57(11.94)	3.22(1.74)		
History of surgery [a]					$\chi^2_{(2)} = 1.48$	0.476
No	11(36.7)	8(25.8)	7(23.3)	26(28.6)		
Yes	19(63.3)	23(74.2)	23(76.7)	65(71.4)		
Take medicine						
hypertension	18(60.0)	22(71.0)	15(50.0)	55(60.4)		
sedative sleeping pills	15(50.0)	9(29.0)	9(30.0)	33(36.3)		
Hyperlipidemia	13(43.3)	9(29.0)	6(20.0)	28(30.8)		
Diabetes mellites	8(26.7)	6(19.4)	5(16.7)	19(20.9)		
Cardiovascular disease	4(13.3)	8(25.8)	5(16.7)	17(18.7)		
Promote cerebral blood circulation	1(3.3)	2(6.5)	7(23.3)	10(11.0)		
Gastrointestinal diseases	1(3.3)	4(12.9)	4(13.3)	9(9.9)		
kidney disease	0(0.0)	2(6.5)	2(6.7)	4(4.4)		
antidepressant	1(3.3)	3(9.7)	2(6.7)	6(6.6)		
traditional Chinese medicine	1(3.3)	1(3.2)	2(6.7)	4(4.4)		
Parkinson's disease	0(0.0)	0(0.0)	2(6.7)	2(2.2)		
Asthma medication	1(3.3)	0(0.0)	1(3.3)	2(2.2)		

Variable	G0 (n = 30) n(%)	G1 (n = 31) n(%)	G2 (n = 30) n(%)	Total (n = 91) n(%)	F/χ^2	*P* value
Anti-inflammatory and analgesic	1(3.3)	0(0.0)	1(3.3)	2(2.2)		
Total number of medications [b]					$F_{(2,88)} = 0.41$	0.960
Range(median)	0-5(2)	0-7(2)	0-6(2)	0-7(2)		
Mean(SD)	2.33(1.24)	2.26(1.57)	2.37(1.69)	2.32(1.50)		

Note: Data are presented as mean ± standard deviation or frequency (%). $^*p$ <0.05

[a] Fisher's exact test; [b] One-way ANOVA

Abbreviations: Control group, G0; Experimental Group 1 received Tongue Strengthening Exercises (TSE) and Chin Tuck Against Resistance Exercise Training (TSE+CTAR), G1; Experimental Group 2 received Chin Tuck Against Resistance Exercise Training (CTAR), G2; Not applicable, NA

Table S2. Effects of Synergistic TSE and CTAR on Swallowing Function (Tongue Strength, Swallowing Pressure, Tongue Endurance, and Lip Strength), Oral Intake, and Cognitive Function

Outcome	G1 (n = 31)	G2 (n = 30)	G0 (n = 30)	G1 (n = 31)	G2 (n = 30)	G0 (n = 30)
**Tongue strength**	**ATS**			**PTS**		
Baseline †	37.4 ± 12.10	37.9 ± 9.68	41.3 ± 11.27	33.8 ± 12.52	37.3 ± 13.78	39.9 ± 12.67
T1	40.0 ± 7.82	41.6 ± 9.96	40.2 ± 11.89	38.7 ± 9.72	43.5 ± 11.61	40.2 ± 11.13
T2	41.9 ± 6.88	42.4 ± 8.75	43.2 ± 10.35	40.2 ± 9.15	45.2 ± 13.23	39.2 ± 13.13
T3	41.3 ± 12.06	44.3 ± 9.85	41.3 ± 11.42	42.6 ± 9.39	44.9 ± 11.25	41.3 ± 12.29
T4	42.3 ± 8.55	41.8 ± 8.18	43.1 ± 9.55	40.7 ± 10.15	43.4 ± 10.81	41.0 ± 12.58
T5	42.9 ± 6.69	43.3 ± 8.49	41.7 ± 11.19	41.7 ± 9.24	45.8 ± 11.51	44.8 ± 11.52
T6	42.5 ± 8.68	44.1 ± 9.78	42.7 ± 9.30	41.7 ± 10.00	37.3 ± 13.78	42.6 ± 13.24
**Tongue Pressure**	**SSP**			**ESP**		
Baseline†	28.9 ± 11.00	27.7 ± 10.60	34.3 ± 11.65	33.7 ± 12.15	31.7 ± 15.21	35.0 ± 11.19
T1	33.5 ± 10.45	31.8 ± 11.62	31.4 ± 12.60	38.3 ± 11.48	35.4 ± 13.62	33.6 ± 10.35
T2	36.4 ± 9.95	33.5 ± 11.59	31.0 ± 9.89	39.6 ± 11.37	39.7 ± 11.81	35.3 ± 9.63
T3	35.4 ± 9.17	35.2 ± 10.71	27.5 ± 10.28	40.5 ± 9.94	42.0 ± 13.15	36.1 ± 12.90
T4	31.0 ± 11.73	33.8 ± 11.93	8.5 ± 10.02	39.5 ± 11.38	39.0 ± 13.94	38.0 ± 8.80
T5	33.3 ± 10.31	33.8 ± 9.95	33.0 ± 11.47	41.8 ± 11.24	42.4 ± 11.42	39.7 ± 11.26
T6	31.9 ± 11.98	34.2 ± 9.98	31.2 ± 10.39	42.0 ± 10.34	43.0 ± 11.86	38.3 ± 11.87
**Tongue Endurance**	**ATE Target Max**			**PTE Target Max**		
Baseline †	42.0 ± 10.34	43.0 ± 11.86	38.3 ± 11.87	32.7 ± 14.66	34.3 ± 14.88	36.9 ± 12.91
T1	36.1 ± 12.74	36.0 ± 12.14	38.3 ± 12.38	41.5 ± 10.28	39.6 ± 12.46	37.6 ± 14.87
T2	38.6 ± 8.09	38.0 ± 12.34	38.5 ± 13.37	40.5 ± 8.79	43.0 ± 12.20	37.8 ± 13.18
T3	40.7 ± 8.78	43.1 ± 11.81	41.0 ± 12.13	40.9 ± 11.40	42.1 ± 11.72	43.6 ± 12.69
T4	41.0 ± 9.41	45.1 ± 11.20	40.3 ± 12.09	39.7 ± 10.02	41.3 ± 13.94	42.3 ± 12.09
T5	40.8 ± 8.76	40.8 ± 11.81	42.0 ± 9.91	40.8 ± 11.06	41.9 ± 12.19	42.9 ± 11.61
T6	39.2 ± 12.96	41.9 ± 11.68	44.3 ± 8.94	40.3 ± 10.80	43.7 ± 12.93	40.0 ± 13.06
**Tongue Endurance**	**ATE Target Sec**			**PTE Target Sec**		
Baseline †	6.1 ± 5.66	4.9 ± 5.17	6.3 ± 8.59	3.8 ± 3.91	3.3 ± 2.96	4.2 ± 5.95
T1	7.3 ± 5.42	6.6 ± 6.57	6.6 ± 10.45	6.2 ± 5.98	4.9 ± 6.74	4.6 ± 6.69
T2	7.8 ± 6.64	7.5 ± 6.76	9.9 ± 9.07	4.2 ± 3.26	4.0 ± 4.44	8.9 ± 9.66
T3	9.1 ± 11.08	7.1 ± 5.98	8.7 ± 8.34	5.7 ± 6.84	5.8 ± 6.27	7.8 ± 6.80
T4	8.3 ± 7.04	8.4 ± 7.73	10.8 ± 10.27	4.8 ± 4.66	4.0 ± 3.91	8.6 ± 9.49
T5	11.010.71	6.3 ± 5.55	11.8 ± 10.06	6.0 ± 5.93	4.5 ± 3.30	8.1 ± 8.56
T6	10.5 ± 10.03	8.6 ± 8.01	11.5 ± 9.95	6.0 ± 4.53	6.1 ± 6.01	7.7 ± 10.95
	**Oral Intake (FOIS)**			**Cognitive Function (MMSE)**		
Baseline †	6.7 ± 0.65	6.4 ± 0.77	6.7 ± 0.65	25.5 ± 3.25	25.4 ± 2.33	24.5 ± 5.47
T1	6.8 ± 0.50	6.5 ± 0.78	6.8 ± 0.50	26.4 ± 2.35	25.2 ± 2.65	23.8 ± 5.87
T2	6.8 ± 0.41	6.5 ± 0.78	6.8 ± 0.41	26.3 ± 2.48	25.3 ± 2.62	23.8 ± 5.87
T3	6.9 ± 0.25	6.5 ± 0.78	6.9 ± 0.25	26.2 ± 2.62	25.3 ± 2.55	23.9 ± 5.83
T4	6.9 ± 0.25	6.5 ± 0.78	6.9 ± 0.25	26.0 ± 2.47	25.3 ± 2.61	23.8 ± 5.95
T5	6.9 ± 0.25	6.7 ± 0.64	6.9 ± 0.25	26.1 ± 2.52	25.5 ± 2.87	23.6 ± 6.02
T6	7.0 ± 0.00	6.7 ± 0.63	7.0 ± 0.00	25.4 ± 3.57	25.2 ± 3.00	24.1 ± 5.81
**Lip Strength**	**LLS**			**RLS**		
Baseline †	24.2 ± 10.49	20.9 ± 6.76	22.8 ± 7.77	24.5 ± 11.51	20.9 ± 7.23	25.5 ± 3.25
T1	24.1 ± 6.12	22.9 ± 6.16	24.4 ± 4.70	22.4 ± 5.40	22.6 ± 7.65	26.4 ± 2.35
T2	25.0 ± 5.79	23.0 ± 6.95	24.8 ± 6.37	23.2 ± 6.38	22.1 ± 6.22	26.3 ± 2.48
T3	24.5 ± 6.88	22.9 ± 6.41	23.0 ± 8.74	22.2 ± 5.12	21.6 ± 7.51	26.2 ± 2.62
T4	24.5 ± 4.66	22.5 ± 7.78	25.9 ± 7.99	24.7 ± 8.02	21.1 ± 7.50	26.0 ± 2.47
T5	23.9 ± 5.15	21.5 ± 6.04	22.6 ± 5.83	21.9 ± 5.13	22.1 ± 6.41	26.1 ± 2.52
T6	23.4 ± 5.17	22.3 ± 5.62	22.3 ± 5.73	21.8 ± 5.28	22.5 ± 5.84	25.4 ± 3.57

Note: Data are presented as mean (standard deviation); $^{*}p < 0.05$; $^{**}p < 0.005$; † one-way analysis of variance.
Abbreviations: G0, control group; G1, experimental group 1: tongue strengthening exercises (TSE) and chin tuck against resistance exercise (CTAR; TSE + CTAR); G2, experimental group 2: CTAR; NA, not applicable.
ATS, anterior tongue strength; PTS, posterior tongue strength;
SSP, saliva swallowing pressure; ESP, effortful swallowing pressure;
ATE-Target Max, anterior tongue endurance target maximum; PTE-Target Max, posterior tongue endurance target maximum;

ATE-Target Sec, anterior tongue endurance target second; PTE-Target Sec, posterior tongue endurance target second;
FOIS, functional oral intake scale; MMSE: Mini-Mental State Examination;
LLS, Left Lip strength; RLS, Right Lip strength;
IOPI: Iowa Oral Performance Instrument
T0 (Baseline – Pre-Initial Training Test), T1 (1-Month Mid-Test), T2 (2-Month Mid-Test), T3 (3-Month Immediate Post-Initial Training Test), T4 (6-Month Follow-Up – 3-Month Post-Initial Training Test), Booster Training initiated right after 3-Month Post-Initial Training Test at T4), T5 (9-Month – 3-Month Immediate Post-Booster Training Test), and T6 (12-Month Follow-Up – 3-Month Post-Booster Training Test)

Table S3. GEE Analysis of Differences between Pretest and Posttest for Anterior Tongue Strength (N=91)

Variable	*B*	SE	95% CI	Waldχ2	*P* value
ATS					
Intercept	41.3	2.0	(37.3, 45.3)	417.1	0.000
Group 2(Exp)[a]	-3.4	2.7	(-8.7, 1.8)	1.7	0.198
Group 1(Exp)[a]	-3.9	2.9	(-9.7, 1.9)	1.7	0.187
Time (6th)[b]	-0.2	1.4	(-3.0, 2.5)	0.0	0.866
Time (5th)[b]	-1.3	1.8	(-4.7, 2.2)	0.5	0.465
Time (4th)[b]	0.2	1.5	(-2.7, 3.0)	0.0	0.912
Time (3rd)[b]	-1.2	1.2	(-3.6, 1.1)	1.0	0.313
Time (2nd)[b]	0.6	1.1	(-1.5, 2.7)	0.3	0.580
Time (1st)[b]	-0.9	1.4	(-3.7, 1.8)	0.4	0.508
Interactions					
Group2 (Exp) x time (6th)[c]	6.3	2.8	(0.8, 11.8)	5.0	0.026*
Group2 (Exp) x time (5th)[c]	6.9	2.9	(1.2, 12.6)	5.6	0.018
Group 2(Exp) x time (4th)[c]	4.0	2.4	(-0.8, 8.8)	2.6	0.106
Group 2(Exp) x time (3rd)[c]	7.8	2.3	(3.2, 12.4)	11.2	0.001**
Group 2(Exp) x time (2nd)[c]	4.1	1.7	(0.7, 7.4)	5.7	0.017*
Group 2(Exp) x time (1st)[c]	5.1	2.0	(1.2, 9.0)	6.4	0.011*
Group1 (Exp) x time (6th)[c]	5.7	2.3	(1.1, 10.2)	5.9	0.015*
Group1 (Exp) x time (5th)[c]	6.8	2.5	(1.9, 11.8)	7.4	0.007**
Group 1(Exp) x time (4th)[c]	4.8	2.5	(-0.1, 9.8)	3.7	0.056
Group 1(Exp) x time (3rd)[c]	6.5	2.5	(1.6, 11.4)	6.8	0.009**
Group 1(Exp) x time (2nd)[c]	4.0	2.2	(-0.3, 8.3)	3.3	0.070
Group 1(Exp) x time (1st)[c]	3.5	2.2	(-0.8, 7.9)	2.5	0.111

Note: "1st": the measurement at 1-month mid-test; "2nd": the measurement at 2-month mid-test, "3rd": the measurement at 3-month immediate post-test, "4th": the measurement at 6-month follow-up, "5th": the measurement at 9-month follow-up, "6th": the measurement at 1-year follow-up, [a] Reference group: control group; [b] Reference group: time (first); [C] Reference group (CON) x time (baseline). $^{*}p<0.05$; $^{**}p<0.01$; $^{***}p<0.001$, Group 1: TSE+CTAR, Group 2: CTAR, Exp: Experimental group; FS: Frailty Status, GEE: generalized estimating equation; ATS: Anterior tongue Strength.

Table S4. GEE Analysis of Differences between Pretest and Posttest for Posterior Tongue Strength (N=91)

Variable	*B*	SE	95% CI	Waldχ2	*P* value
PTS					
Intercept	39.9	2.3	(35.5, 44.4)	308.5	0.000
Group 2(Exp)[a]	-2.6	3.4	(-9.2, 3.9)	0.6	0.432
Group 1(Exp)[a]	-6.1	3.2	(-12.3, 0.1)	3.7	0.053
Time (6th)[b]	1.1	2.0	(-2.8, 5.0)	0.3	0.578
Time (5th)[b]	3.4	1.4	(0.6, 6.1)	5.8	0.016
Time (4th)[b]	-0.4	2.0	(-4.4, 3.5)	0.0	0.824
Time (3rd)[b]	0.7	1.9	(-3.0, 4.3)	0.1	0.715
Time (2nd)[b]	-1.8	1.7	(-5.2, 1.6)	1.1	0.288
Time (1st)[b]	0.5	1.2	(-1.8, 2.7)	0.2	0.693
Interactions					
Group2 (Exp) x time (6th)[c]	5.0	3.5	(1.9, 11.9)	2.0	0.155
Group2 (Exp) x time (5th)[c]	5.2	2.1	(1.1, 9.3)	6.0	0.014*
Group2 (Exp) x time (4th)[c]	6.6	3.1	(0.5, 12.6)	4.5	0.034*
Group2 (Exp) x time (3rd)[c]	7.0	2.3	(2.4, 11.5)	8.9	0.003**
Group2 (Exp) x time (2nd)[c]	9.7	2.4	(5.0, 14.5)	16.4	0.001***
Group2 (Exp) x time (1st)[c]	6.8	1.7	(3.5, 10.1)	16.5	0.001***
Group1 (Exp) x time (6th)[c]	7.3	2.5	(2.3, 12.2)	8.3	0.004**
Group1 (Exp) x time (5th)[c]	4.8	2.1	(0.7, 8.8)	5.3	0.022*
Group1 (Exp) x time (4th)[c]	7.6	2.8	(2.1, 13.0)	7.3	0.007**
Group1 (Exp) x time (3rd)[c]	8.4	2.7	(3.0, 13.7)	9.5	0.002**
Group1 (Exp) x time (2nd)[c]	8.4	2.4	(3.6, 13.2)	11.9	0.001***
Group1 (Exp) x time (1st)[c]	4.4	1.9	(0.6, 8.2)	5.3	0.022*

Note: "1st": the measurement at 1-month mid-test; "2nd": the measurement at 2-month mid-test, "3rd": the measurement at 3-month immediate post-test, "4th": the measurement at 6-month follow-up, "5th": the measurement at 9-month follow-up, "6th": the measurement at 1-year follow-up, [a] Reference group: control group; [b] Reference group: time (first); [C] Reference group (CON) x time (baseline). $^{*}p<0.05$; $^{**}p<0.01$; $^{***}p<0.001$, Group 1: TSE+CTAR, Group 2CTAR, Exp: Experimental group; FS: Frailty Status, GEE: generalized estimating equation; PTS: Posterior tongue Strength

Table S5. GEE Analysis of Differences between Pretest and Posttest for Saliva Swallowing Pressure (N=91)

Variable	*B*	SE	95% CI	Waldχ2	*P* value
SSP					
Intercept	34.3	2.1	(30.2, 38.4)	269.4	0.000
Group 2(Exp)[a]	-6.7	2.8	(-12.2 -1.1)	5.6	0.018
Group 1(Exp)[a]	-5.5	2.9	(-11.0, 0.1)	3.7	0.056
Time (6th)[b]	-3.9	1.9	(-7.6, -0.3)	4.4	0.036
Time (5th)[b]	-2.1	2.0	(-6.1, 1.8)	1.1	0.288
Time (4th)[b]	-6.6	2.4	(-11.3, -1.9)	7.6	0.006
Time (3rd)[b]	-6.9	1.8	(-10.4, -3.4)	14.6	0.000
Time (2nd)[b]	-3.3	1.9	(-7.1, 0.4)	3.0	0.081
Time (1st)[b]	-2.9	1.6	(-5.9, 0.2)	3.4	0.064
Interactions					
Group 2 (Exp) x time (6th)[c]	9.0	2.5	(4.2, 13.9)	13.6	0.001***
Group 2 (Exp) x time (5th)[c]	7.3	2.8	(1.9, 12.7)	7.0	0.008**
Group 2 (Exp) x time (4th)[c]	11.8	2.9	(6.1, 17.5)	16.4	0.001***
Group 2 (Exp) x time (3rd)[c]	13.4	2.6	(8.3, 18.5)	26.5	0.001***
Group 2 (Exp) x time (2nd)[c]	8.1	2.9	(2.4, 13.9)	7.8	0.005**
Group 2 (Exp) x time (1st)[c]	7.6	2.4	(3.0, 12.2)	10.3	0.001***
Group 1 (Exp) x time (6th)[c]	7.2	2.9	(1.5, 12.8)	6.1	0.014*
Group 1 (Exp) x time (5th)[c]	6.5	2.9	(0.9, 12.1)	5.1	0.024*
Group 1 (Exp) x time (4th)[c]	8.6	3.0	(2.8, 14.5)	8.3	0.004**
Group 1 (Exp) x time (3rd)[c]	13.3	2.5	(8.5, 18.2)	29.4	0.001***
Group 1 (Exp) x time (2nd)[c]	10.8	2.4	(6.1, 15.6)	20.0	0.001***
Group 1 (Exp) x time (1st)[c]	7.5	2.1	(3.5, 11.6)	13.5	0.001***

Note: "1st": the measurement at 1-month mid-test; "2nd": the measurement at 2-month mid-test, "3rd": the measurement at 3-month immediate post-test, "4th": the measurement at 6-month follow-up, "5th": the measurement at 9-month follow-up, "6th": the measurement at 1-year follow-up, [a] Reference group: control group; [b] Reference group: time (first); [C] Reference group (CON) x time (baseline). $^{*}p<0.05$; $^{**}p<0.01$; $^{***}p<0.001$, Group 1: TSE+CTAR, Group 2: CTAR, Exp: Experimental group; FS: Frailty Status, GEE: generalized estimating equation; SSP: Saliva swallowing pressure

Table S6. GEE Analysis of Differences between Pretest and Posttest for Effortful Swallowing Pressure (N=91)

Variable	*B*	SE	95% CI	Wald$\chi 2$	*P value*
ESP					
Intercept	35.0	2.0	(31.0, 38.9)	303.4	0.000
Group 2(Exp)[a]	-3.2	3.4	(-9.9, 3.4)	0.9	0.339
Group 1(Exp)[a]	-1.3	2.9	(-7.0, 4.5)	0.2	0.668
Time (6th)[b]	1.9	2.5	(-3.0, 6.9)	0.6	0.450
Time (5th)[b]	3.3	2.4	(-1.5, 8.1)	1.8	0.174
Time (4th)[b]	1.7	1.9	(-2.1, 5.4)	0.7	0.387
Time (3rd)[b]	0.6	2.2	(-3.6, 4.8)	0.1	0.776
Time (2nd)[b]	-0.3	1.8	(-3.8, 3.2)	0.0	0.864
Time (1st)[b]	-1.2	1.9	(-4.9, 2.6)	0.4	0.545
Interactions					
Group2 (Exp) x time (6th)[c]	7.4	3.2	(1.1, 13.6)	5.4	0.020*
Group2 (Exp) x time (5th)[c]	5.8	3.1	(-0.3, 11.8)	3.5	0.060
Group 2(Exp) x time (4th)[c]	4.0	2.8	(-1.6, 9.5)	2.0	0.160
Group 2(Exp) x time (3rd)[c]	8.0	3.0	(2.1, 13.9)	7.2	0.007**
Group 2(Exp) x time (2nd)[c]	6.6	2.4	(1.9, 11.4)	7.5	0.006**
Group 2(Exp) x time (1st)[c]	5.5	2.3	(1.0, 10.0)	5.7	0.017*
Group1 (Exp) x time (6th)[c]	6.8	3.2	(0.5, 13.1)	4.5	0.034*
Group1 (Exp) x time (5th)[c]	4.8	3.1	(-1.3, 10.9)	2.4	0.120
Group 1(Exp) x time (4th)[c]	4.2	2.9	(-1.4, 9.8)	2.2	0.138
Group 1(Exp) x time (3rd)[c]	6.2	2.8	(0.7, 11.7)	4.9	0.027*
Group 1(Exp) x time (2nd)[c]	6.3	2.6	(1.2, 11.4)	5.8	0.016*
Group 1(Exp) x time (1st)[c]	5.7	2.5	(0.9, 10.6)	5.4	0.020*

Note: "1st": the measurement at 1-month mid-test; "2nd": the measurement at 2-month mid-test, "3rd": the measurement at 3-month immediate post-test, "4th": the measurement at 6-month follow-up, "5th": the measurement at 9-month follow-up, "6th": the measurement at 1-year follow-up, [a] Reference group: control group; [b] Reference group: time (first); [C] Reference group (CON) x time (baseline). $^{*}p$ <0.05; $^{**}p$ < 0.01; $^{***}p$ < 0.001, Group 1: TSE+CTAR, Group 2: CTAR, Exp: Experimental group; GEE: generalized estimating equation; ESP, effortful swallowing pressure

Table S7. GEE Analysis of Differences between Pretest and Posttest for Anterior Tongue Endurance Target Maximum (N=91)

Variable	*B*	SE	95% CI	Waldχ2	*P* value
ATE-Target Max					
Intercept	38.3	2.2	(33.9 42.7)	296.8	0.000
Group 2(Exp)[a]	-2.3	3.1	-(8.4 3.8)	0.5	0.468
Group 1(Exp)[a]	-2.2	3.2	-(8.4 4.0)	0.5	0.482
Time (6th)[b]	4.2	1.7	(0.9 7.5)	6.2	0.012
Time (5th)[b]	4.3	1.5	(1.4 7.2)	8.7	0.003
Time (4th)[b]	2.1	2.1	-(2.1 6.3)	1.0	0.329
Time (3rd)[b]	0.7	1.6	-(2.5 3.9)	0.2	0.671
Time (2nd)[b]	1.4	2.2	-(3.0 5.8)	0.4	0.534
Time (1st)[b]	0.8	1.7	-(2.6 4.2)	0.2	0.644
Interactions					
Group2 (Exp) x time (6th)[c]	1.8	2.8	-(3.7 7.2)	0.4	0.525
Group2 (Exp) x time (5th)[c]	0.3	2.4	-(4.3 5.0)	0.0	0.889
Group 2(Exp) x time (4th)[c]	1.4	3.0	-(4.5 7.3)	0.2	0.639
Group 2(Exp) x time (3rd)[c]	7.1	2.7	(1.8 12.4)	6.9	0.009
Group 2(Exp) x time (2nd)[c]	4.4	3.0	-(1.5 10.3)	2.2	0.140
Group 2(Exp) x time (1st)[c]	2.8	2.6	-(2.4 7.9)	1.1	0.292
Group1 (Exp) x time (6th)[c]	0.7	2.8	-(4.8 6.2)	0.1	0.806
Group1 (Exp) x time (5th)[c]	-1.0	2.7	-(6.3 4.2)	0.2	0.695
Group 1(Exp) x time (4th)[c]	2.8	3.2	-(3.5 9.0)	0.8	0.382
Group 1(Exp) x time (3rd)[c]	4.4	2.9	-(1.4 10.1)	2.2	0.135
Group 1(Exp) x time (2nd)[c]	3.4	3.2	-(2.9 9.7)	1.1	0.295
Group 1(Exp) x time (1st)[c]	1.7	2.5	-(3.3 6.7)	0.4	0.509

Note: "1st": the measurement at 1-month mid-test; "2nd": the measurement at 2-month mid-test, "3rd": the measurement at 3-month immediate post-test, "4th": the measurement at 6-month follow-up, "5th": the measurement at 9-month follow-up, "6th": the measurement at 1-year follow-up, [a] Reference group: control group; [b] Reference group: time (first); [C] Reference group (CON) x time (baseline). $^{*}p<0.05$; $^{**}p<0.01$; $^{***}p<0.001$, Group 1: TSE+CTAR, Group 2: CTAR, Exp: Experimental group; GEE: generalized estimating equation; ATE-Target Max, anterior tongue endurance target maximum

Table S8. GEE Analysis of Differences between Pretest and Posttest for Posterior Tongue Endurance Target Maximum (N=91)

Variable	*B*	SE	95% CI	Wald$\chi 2$	*P* value
PTE-Target Max					
Intercept	36.9	2.3	(32.4 41.4)	253.3	0.000
Group 2(Exp)[a]	-2.6	3.5	-(9.5 4.4)	0.5	0.466
Group 1(Exp)[a]	-4.2	3.5	-(11.0 2.6)	1.5	0.224
Time (6th)[b]	1.0	3.0	-(4.8 6.8)	0.1	0.736
Time (5th)[b]	3.9	2.2	-(0.5 8.3)	3.0	0.083
Time (4th)[b]	3.3	2.6	-(1.7 8.3)	1.7	0.198
Time (3rd)[b]	5.4	2.5	(0.5 10.3)	4.7	0.031
Time (2nd)[b]	-0.6	2.5	-(5.6 4.3)	0.1	0.806
Time (1st)[b]	1.4	2.6	-(3.8 6.6)	0.3	0.599
Interactions					
Group2 (Exp) x time (6th)[c]	7.6	4.1	-(0.5 15.7)	3.4	0.066
Group2 (Exp) x time (5th)[c]	3.4	3.3	-(3.0 9.8)	1.1	0.294
Group 2(Exp) x time (4th)[c]	3.5	3.4	-(3.2 10.1)	1.0	0.310
Group 2(Exp) x time (3rd)[c]	2.2	3.2	-(4.0 8.3)	0.5	0.492
Group 2(Exp) x time (2nd)[c]	9.0	3.3	(2.5 15.5)	7.4	0.006
Group 2(Exp) x time (1st)[c]	4.9	3.4	-(1.7 11.6)	2.1	0.145
Group1 (Exp) x time (6th)[c]	7.2	3.8	-(0.2 14.6)	3.6	0.057
Group1 (Exp) x time (5th)[c]	4.6	3.0	-(1.3 10.5)	2.4	0.123
Group 1(Exp) x time (4th)[c]	4.1	3.5	-(2.8 11.0)	1.4	0.241
Group 1(Exp) x time (3rd)[c]	3.1	3.5	-(3.7 9.9)	0.8	0.369
Group 1(Exp) x time (2nd)[c]	8.8	3.5	(2.0 15.7)	6.3	0.012
Group 1(Exp) x time (1st)[c]	7.5	3.5	(0.6 14.3)	4.5	0.033

Note: “1st”: the measurement at 1-month mid-test; “2nd”: the measurement at 2-month mid-test, “3rd”: the measurement at 3-month immediate post-test, “4th”: the measurement at 6-month follow-up, “5th”: the measurement at 9-month follow-up, “6th”: the measurement at 1-year follow-up, [a] Reference group: control group; [b] Reference group: time (first); [C] Reference group (CON) x time (baseline). $^{*}p<0.05$; $^{**}p<0.01$; $^{***}p<0.001$, Group 1: TSE+CTAR, Group 2: CTAR, Exp: Experimental group; GEE: generalized estimating equation; PTE-Target Max, posterior tongue endurance target maximum

Table S9. GEE Analysis of Differences between Pretest and Posttest for Anterior Tongue Endurance Target Second (N=91)

Variable	*B*	SE	95% CI	Waldχ2	*P* value
ATE-Target Sec					
Intercept	6.3	1.5	(3.3 9.3)	16.6	0.000
Group 2(Exp)[a]	-1.4	1.8	-(4.9 2.2)	0.6	0.449
Group 1(Exp)[a]	-0.1	1.8	-(3.7 3.5)	0.0	0.937
Time (6th)[b]	4.3	2.2	(0.0 8.6)	3.8	0.052
Time (5th)[b]	4.5	2.3	(0.0 9.1)	3.8	0.050
Time (4th)[b]	3.5	2.1	-(0.6 7.6)	2.9	0.090
Time (3rd)[b]	1.7	2.1	-(2.4 5.9)	0.7	0.418
Time (2nd)[b]	3.0	2.0	-(1.0 6.9)	2.2	0.142
Time (1st)[b]	0.8	1.4	-(1.9 3.5)	0.3	0.558
Interactions					
Group2 (Exp) x time (6th)[c]	-1.0	2.7	-(6.3 4.3)	0.1	0.725
Group2 (Exp) x time (5th)[c]	-3.4	2.7	-(8.6 1.9)	1.6	0.212
Group 2(Exp) x time (4th)[c]	-0.3	2.8	-(5.8 5.1)	0.0	0.909
Group 2(Exp) x time (3rd)[c]	0.3	2.5	-(4.6 5.1)	0.0	0.910
Group 2(Exp) x time (2nd)[c]	-0.6	2.5	-(5.6 4.3)	0.1	0.799
Group 2(Exp) x time (1st)[c]	1.6	2.3	-(2.9 6.2)	0.5	0.480
Group1 (Exp) x time (6th)[c]	0.1	2.7	-(5.2 5.4)	0.0	0.976
Group1 (Exp) x time (5th)[c]	0.2	3.0	-(5.6 6.1)	0.0	0.934
Group 1(Exp) x time (4th)[c]	-1.4	2.4	-(6.1 3.3)	0.3	0.559
Group 1(Exp) x time (3rd)[c]	1.2	3.0	-(4.6 7.0)	0.2	0.691
Group 1(Exp) x time (2nd)[c]	-1.3	2.3	-(5.8 3.2)	0.3	0.563
Group 1(Exp) x time (1st)[c]	0.3	1.7	-(3.0 3.7)	0.0	0.852

Note: "1st": the measurement at 1-month follow-up; "2nd": the measurement at 2-month follow-up, "3rd": the measurement at 3-month follow-up, "4th": the measurement at 6-month follow-up, "5th": the measurement at 9-month follow-up, "6th": the measurement at 1-year follow-up, [a] Reference group: control group; [b] Reference group: time (first); [C] Reference group (CON) x time (baseline). $^{*}p < 0.05$; $^{**}p < 0.01$; $^{***}p < 0.001$, Group 1: TSE+CTAR, Group 2: CTAR, Exp: Experimental group; GEE: generalized estimating equation; ATE-Target Sec, anterior tongue endurance target second

Table S10. GEE Analysis of Differences between Pretest and Posttest for Posterior Tongue Endurance Target Second (N=91)

Variable	*B*	SE	95% CI	Waldχ2	*P* value
PTE-Target Sec					
Intercept	4.2	1.1	(2.1 6.3)	15.8	0.000
Group 2(Exp)[a]	-0.9	1.2	-(3.2 1.4)	0.6	0.452
Group 1(Exp)[a]	-0.4	1.3	-(2.9 2.1)	0.1	0.728
Time (6th)[b]	3.1	2.5	-(1.9 8.1)	1.5	0.225
Time (5th)[b]	3.5	2.3	-(0.9 8.0)	2.4	0.118
Time (4th)[b]	4.1	2.0	(0.2 8.0)	4.2	0.041
Time (3rd)[b]	3.4	1.8	-(0.2 6.9)	3.5	0.063
Time (2nd)[b]	4.4	2.1	(0.3 8.4)	4.4	0.036
Time (1st)[b]	0.7	1.6	-(2.4 3.8)	0.2	0.655
Interactions					
Group2 (Exp) x time (6th)[c]	-0.3	2.8	-(5.7 5.1)	0.0	0.917
Group2 (Exp) x time (5th)[c]	-2.4	2.4	-(7.2 2.4)	1.0	0.330
Group 2(Exp) x time (4th)[c]	-3.4	2.1	-(7.5 0.7)	2.6	0.105
Group 2(Exp) x time (3rd)[c]	-0.9	2.1	-(5.1 3.3)	0.2	0.663
Group 2(Exp) x time (2nd)[c]	-3.7	2.2	-(8.1 0.7)	2.7	0.102
Group 2(Exp) x time (1st)[c]	1.3	2.1	-(2.8 5.4)	0.4	0.526
Group1 (Exp) x time (6th)[c]	-1.0	2.7	-(6.3 4.3)	0.1	0.722
Group1 (Exp) x time (5th)[c]	-1.4	2.5	-(6.2 3.5)	0.3	0.585
Group 1(Exp) x time (4th)[c]	-3.0	2.2	-(7.4 1.4)	1.8	0.178
Group 1(Exp) x time (3rd)[c]	-1.5	2.2	-(5.8 2.8)	0.5	0.502
Group 1(Exp) x time (2nd)[c]	-3.9	2.2	-(8.3 0.5)	3.1	0.081
Group 1(Exp) x time (1st)[c]	1.7	1.9	-(2.1 5.4)	0.8	0.380

Note: “1st”: the measurement at 1-month mid-test; “2nd”: the measurement at 2-month mid-test, “3rd”: the measurement at 3-month immediate post-test, “4th”: the measurement at 6-month follow-up, “5th”: the measurement at 9-month follow-up, “6th”: the measurement at 1-year follow-up, [a] Reference group: control group; [b] Reference group: time (first); [C] Reference group (CON) x time (baseline). $^{*}p<0.05$; $^{**}p < 0.01$; $^{***}p < 0.001$, Group 1: TSE+CTAR, Group 2: CTAR, Exp: Experimental group; GEE: generalized estimating equation; PTE-Target Sec, posterior tongue endurance target second

Table S11. GEE Analysis of Differences between Pretest and Posttest for Left Lip Strength (N=91)

Variable	*B*	SE	95% CI	Waldχ2	*P* value
LLS					
Intercept	22.8	1.4	(0.0, 25.5)	266.2	0.000
Group 2 (Exp)[a]	-1.8	1.8	(-5.4, 1.8)	1.0	0.324
Group 1 (Exp)[a]	1.4	2.3	(-3.1, 6.0)	0.4	0.541
Time (6th)[b]	-0.5	1.1	(-2.7, 1.6)	0.2	0.637
Time (5th)[b]	2.8	2.1	(-1.4, 6.9)	1.7	0.191
Time (4th)[b]	1.3	1.6	(-1.8, 4.5)	0.7	0.407
Time (3rd)[b]	1.8	1.3	(-0.8, 4.4)	1.8	0.178
Time (2nd)[b]	1.3	1.3	(-1.4, 3.9)	0.9	0.347
Time (1st)[b]	-0.2	1.0	(-2.1, 1.8)	0.0	0.870
Interactions					
Group2 (Exp) x time (6th)[c]	0.9	1.7	(-2.5, 4.2)	0.3	0.610
Group2 (Exp) x time (5th)[c]	-1.1	2.7	(-6.4, 4.1)	0.2	0.669
Group2 (Exp) x time (4th)[c]	0.6	2.2	(-3.7, 5.0)	0.1	0.773
Group2 (Exp) x time (3rd)[c]	0.3	1.8	(-3.2, 3.9)	0.0	0.853
Group2 (Exp) x time (2nd)[c]	0.8	2.1	(-3.3, 4.8)	0.1	0.715
Group2 (Exp) x time (1st)[c]	1.3	1.7	(-2.1, 4.6)	0.5	0.460
Group1 (Exp) x time (6th)[c]	0.2	2.2	(-4.1, 4.5)	0.0	0.918
Group1 (Exp) x time (5th)[c]	-2.4	2.8	(-7.9, 3.1)	0.7	0.398
Group1 (Exp) x time (4th)[c]	-1.0	2.5	(-5.9, 4.0)	0.1	0.707
Group1 (Exp) x time (3rd)[c]	-0.9	2.5	(-5.9, 4.1)	0.1	0.718
Group1 (Exp) x time (2nd)[c]	-1.2	2.3	(-5.8, 3.4)	0.3	0.605
Group1 (Exp) x time (1st)[c]	-0.7	2.1	(-4.7, 3.4)	0.1	0.751

Note: "1st": the measurement at 1-month mid-test; "2nd": the measurement at 2-month mid-test, "3rd": the measurement at 3-month immediate post-test, "4th": the measurement at 6-month follow-up, "5th": the measurement at 9-month follow-up, "6th": the measurement at 1-year follow-up, a Reference group: control group; b Reference group: time (first); C Reference group (CON) x time (baseline). *p <0.05; **p < 0.01; ***p < 0.001, Group 1: TSE+CTAR, Group 2: CTAR, Exp: Experimental group; FS: Frailty Status, GEE: generalized estimating equation; LLS: Left Lip Strength

Table S12. GEE Analysis of Differences between Pretest and Posttest for Right Left Strength (N=91)

Variable	*B*	SE	95% CI	Waldχ2	*P* value
RLS					
Intercept	23.3	1.0	(21.4, 25.3)	548.2	0.000
Group 2(Exp)[a]	-2.4	1.6	(-5.6, 0.8)	2.2	0.141
Group 1(Exp)[a]	1.2	2.3	(-3.2, 5.6)	0.3	0.599
Time (6th)[b]	-0.7	1.0	(-2.7, 1.3)	0.4	0.507
Time (5th)[b]	1.6	1.2	(-0.8, 4.0)	1.8	0.186
Time (4th)[b]	-0.1	0.7	(-1.6, 1.3)	0.0	0.858
Time (3rd)[b]	1.0	0.9	(-0.8, 2.7)	1.2	0.271
Time (2nd)[b]	0.2	1.1	(-1.9, 2.3)	0.0	0.874
Time (1st)[b]	-0.4	0.6	(-1.5, 0.7)	0.5	0.486
Interactions					
Group 2 (Exp) x time (6th)[c]	1.7	1.5	(-1.3, 4.7)	1.3	0.258
Group 2 (Exp) x time (5th)[c]	-0.6	1.5	(-3.6, 2.4)	0.2	0.685
Group 2 (Exp) x time (4th)[c]	0.2	1.4	(-2.5, 2.9)	0.0	0.902
Group 2 (Exp) x time (3rd)[c]	-0.5	1.4	(-3.3, 2.3)	0.1	0.732
Group 2 (Exp) x time (2nd)[c]	0.9	1.5	(-2.0, 3.7)	0.4	0.553
Group 2 (Exp) x time (1st)[c]	2.1	1.1	(-0.1, 4.2)	3.4	0.063
Group 1 (Exp) x time (6th)[c]	-1.9	2.5	(-6.8, 3.0)	0.6	0.442
Group 1 (Exp) x time (5th)[c]	-4.1	2.4	(-8.9, 0.6)	2.9	0.088
Group 1 (Exp) x time (4th)[c]	0.4	2.7	(-4.9, 5.6)	0.0	0.896
Group 1 (Exp) x time (3rd)[c]	-3.2	2.2	(-7.5, 1.2)	2.1	0.150
Group 1 (Exp) x time (2nd)[c]	-1.4	2.4	(-6.2, 3.4)	0.3	0.563
Group 1 (Exp) x time (1st)[c]	-1.7	2.0	(-5.6, 2.2)	0.7	0.402

Note: “1st”: the measurement at 1-month mid-test; “2nd”: the measurement at 2-month mid-test, “3rd”: the measurement at 3-month immediate post-test, “4th”: the measurement at 6-month follow-up, “5th”: the measurement at 9-month follow-up, “6th”: the measurement at 1-year follow-up, [a] Reference group: control group; [b] Reference group: time (first); [C] Reference group (CON) x time (baseline). $^{*}p<0.05$; $^{**}p<0.01$; $^{***}p<0.001$, Group 1: TSE+CTAR, Group 2: CTAR, Exp: Experimental group; FS: Frailty Status, GEE: generalized estimating equation; RLS: Right Lip Strength

Table S13. GEE Analysis of Differences between Pretest and Posttest for Oral Intake (N=91)

Variable	*B*	SE	95% CI	Waldχ2	*P* value
FOIS					
Intercept	6.7	0.1	(6.4 6.9)	2727.3	0.000
Group 2(Exp)[a]	-0.3	0.2	-(0.6 0.1)	2.0	0.156
Group 1(Exp)[a]	0.0	0.2	-(0.3 0.3)	0.0	0.950
Time (6th)[b]	0.3	0.1	(0.1 0.6)	6.2	0.013
Time (5th)[b]	0.3	0.1	(0.1 0.6)	6.0	0.014
Time (4th)[b]	0.3	0.1	(0.1 0.6)	5.8	0.016
Time (3rd)[b]	0.2	0.1	(0.0 0.4)	2.7	0.099
Time (2nd)[b]	0.2	0.1	(0.0 0.4)	2.7	0.100
Time (1st)[b]	0.2	0.1	(0.0 0.4)	2.7	0.102
Interactions					
Group2 (Exp) x time (6th)[c]	-0.1	0.1	-(0.4 0.2)	0.3	0.599
Group2 (Exp) x time (5th)[c]	-0.1	0.1	-(0.4 0.2)	0.3	0.564
Group 2(Exp) x time (4th)[c]	-0.2	0.1	-(0.5 0.1)	2.4	0.120
Group 2(Exp) x time (3rd)[c]	-0.2	0.1	-(0.4 0.1)	1.4	0.244
Group 2(Exp) x time (2nd)[c]	-0.2	0.1	-(0.5 0.0)	2.7	0.101
Group 2(Exp) x time (1st)[c]	-0.2	0.1	-(0.5 0.0)	3.0	0.081
Group1 (Exp) x time (6th)[c]	0.0	0.2	-(0.3 0.3)	0.0	0.933
Group1 (Exp) x time (5th)[c]	-0.1	0.2	-(0.4 0.3)	0.1	0.772
Group 1(Exp) x time (4th)[c]	0.0	0.2	-(0.4 0.3)	0.1	0.796
Group 1(Exp) x time (3rd)[c]	0.1	0.2	-(0.3 0.4)	0.1	0.725
Group 1(Exp) x time (2nd)[c]	-0.1	0.1	-(0.4 0.2)	0.3	0.615
Group 1(Exp) x time (1st)[c]	-0.1	0.1	-(0.4 0.2)	0.5	0.458

Note: "1st": the measurement at 1-month mid-test; "2nd": the measurement at 2-month mid-test, "3rd": the measurement at 3-month immediate post-test, "4th": the measurement at 6-month follow-up, "5th": the measurement at 9-month follow-up, "6th": the measurement at 1-year follow-up, [a] Reference group: control group; [b] Reference group: time (first); [C] Reference group (CON) x time (baseline). $^{*}p<0.05$; $^{**}p<0.01$; $^{***}p<0.001$, Group 1: TSE+CTAR, Group 2:CTAR, Exp: Experimental group; FS: Frailty Status, GEE: generalized estimating equation; FOIS: functional oral intake scale

Supplemental Table 14 GEE Analysis of Differences between Pretest and Posttest for Cognitive Function (N=91)

**Variable**	***B***	**SE**	**95% CI**		**Waldχ2**	***P* value**
MMSE						
Intercept	25.5	0.6	(24.4	26.6)	1915.6	0.000
Group 2(Exp)[a]	-1.0	1.1	-(3.3	1.2)	0.8	0.365
Group 1(Exp)[a]	-0.1	0.7	-(1.5	1.3)	0.0	0.910
Time (6th)[b]	-1.0	0.7	-(2.3	0.3)	2.3	0.126
Time (5th)[b]	-0.4	0.3	-(1.0	0.2)	1.4	0.244
Time (4th)[b]	-0.4	0.3	-(1.0	0.2)	1.7	0.186
Time (3rd)[b]	-0.2	0.3	-(0.7	0.4)	0.4	0.546
Time (2nd)[b]	0.0	0.2	-(0.4	0.3)	0.0	0.891
Time (1st)[b]	0.1	0.1	-(0.2	0.4)	0.5	0.460
Interactions						
Group2 (Exp) x time (6th)[c]	0.7	0.9	-(1.1	2.5)	0.6	0.440
Group2 (Exp) x time (5th)[c]	0.0	0.5	-(1.0	1.0)	0.0	0.989
Group 2(Exp) x time (4th)[c]	0.2	0.5	-(0.8	1.1)	0.1	0.732
Group 2(Exp) x time (3rd)[c]	0.1	0.4	-(0.7	0.9)	0.0	0.828
Group 2(Exp) x time (2nd)[c]	-0.1	0.3	-(0.8	0.5)	0.2	0.670
Group 2(Exp) x time (1st)[c]	-0.3	0.3	-(0.9	0.3)	0.8	0.378
Group1 (Exp) x time (6th)[c]	0.8	0.7	-(0.6	2.2)	1.1	0.289
Group1 (Exp) x time (5th)[c]	0.4	0.4	-(0.4	1.2)	0.9	0.348
Group 1(Exp) x time (4th)[c]	0.2	0.4	-(0.6	0.9)	0.2	0.680
Group 1(Exp) x time (3rd)[c]	-0.1	0.4	-(0.7	0.6)	0.0	0.876
Group 1(Exp) x time (2nd)[c]	-0.2	0.3	-(0.7	0.4)	0.4	0.550
Group 1(Exp) x time (1st)[c]	-0.4	0.2	-(0.9	0.1)	2.1	0.144

Note: "1st": the measurement at 1-month mid-test; "2nd": the measurement at 2-month mid-test, "3rd": the measurement at 3-month immediate post-test, "4th": the measurement at 6-month follow-up, "5th": the measurement at 9-month follow-up, "6th": the measurement at 1-year follow-up, a Reference group: control group; b Reference group: time (first); C Reference group (CON) x time (baseline). *p <0.05; **p < 0.01; ***p < 0.001, Group 1: TSE+CTAR, Group 2: CTAR, Exp: Experimental group; GEE: generalized estimating equation; MMSE: Mini-Mental State Examination.
